# Defining the Risk of Zika and Chikungunya Virus Transmission in Human Population Centers of the Eastern United States

**DOI:** 10.1371/journal.pntd.0005255

**Published:** 2017-01-17

**Authors:** Carrie A. Manore, Richard S. Ostfeld, Folashade B. Agusto, Holly Gaff, Shannon L. LaDeau

**Affiliations:** 1 Center for Computational Science Tulane University New Orleans, LA, United States of America; 2 Theoretical Biology and Biophysics Los Alamos National Laboratory Los Alamos, NM, United States of America; 3 New Mexico Consortium, Suite 301 Los Alamos, NM, United States of America; 4 Cary Institute of Ecosystem Studies Box AB, 2801 Sharon Turnpike Millbrook, NY United States of America; 5 Department of Ecology and Evolutionary Biology University of Kansas Haworth Hall Lawrence, Kansas, United States of America; 6 Department of Biological Sciences Old Dominion University Norfolk, VA, United States of America; 7 Mathematics, Statistics and Computer Science University of KwaZulu-Natal Durban, South Africa; Santa Fe Institute, UNITED STATES

## Abstract

The recent spread of mosquito-transmitted viruses and associated disease to the Americas motivates a new, data-driven evaluation of risk in temperate population centers. Temperate regions are generally expected to pose low risk for significant mosquito-borne disease; however, the spread of the Asian tiger mosquito (*Aedes albopictus*) across densely populated urban areas has established a new landscape of risk. We use a model informed by field data to assess the conditions likely to facilitate local transmission of chikungunya and Zika viruses from an infected traveler to *Ae*. *albopictus* and then to other humans in USA cities with variable human densities and seasonality. Mosquito-borne disease occurs when specific combinations of conditions maximize virus-to-mosquito and mosquito-to-human contact rates. We develop a mathematical model that captures the epidemiology and is informed by current data on vector ecology from urban sites. The model demonstrates that under specific but realistic conditions, fifty-percent of introductions by infectious travelers to a high human, high mosquito density city could initiate local transmission and 10% of the introductions could result in 100 or more people infected. Despite the propensity for *Ae*. *albopictus* to bite non-human vertebrates, we also demonstrate that local virus transmission and human outbreaks may occur when vectors feed from humans even just 40% of the time. Inclusion of human behavioral changes and mitigations were not incorporated into the models and would likely reduce predicted infections. This work demonstrates how a conditional series of non-average events can result in local arbovirus transmission and outbreaks of human disease, even in temperate cities.

## Author Summary

Zika and chikungunya viruses are transmitted by *Aedes* mosquitoes, including *Ae*. *albopictus*, which is abundant in many temperate cities. While disease risk is lower in temperate regions where viral amplification cannot build across years, there is significant potential for localized disease outbreaks in urban populations. We use a model informed by field data to assess the conditions likely to facilitate local transmission of virus from an infected traveler to *Ae*. *albopictus* and then to other humans in USA cities with variable human densities and seasonality. The model demonstrates that up to 50% of infectious travelers returning to the U.S. could initiate local transmission in temperate cities if are infectious and are exposed to high mosquito densities. This work highlights the need for high-resolution spatial data on *Ae*. *albopictus* density, biting behavior, and seasonality to better understand, predict and manage arboviral transmission risk in temperate cities.

## Introduction

The Asian tiger mosquito (*Aedes albopictus*) is a global nuisance, with self-sustaining populations established on nearly every continent. Like its relative, *Ae*. *aegypti*, the Asian tiger mosquito is a day-time biter and lays eggs that are resistant to drought. In its native range, the juveniles develop in water-holding tree holes and emerging adult females feed opportunistically on vertebrate species in the surrounding sylvan habitats. Limited vagility of adult mosquitoes restricts natural dispersal distances to a few hundred meters [1,2], but international trade and travel has dispersed the species well beyond its native forests of southeast Asia to urban and peri-urban landscapes throughout the Americas and Europe in the 1980s and Africa in the 1990s [3,4]. Similar to the earlier invasion by *Ae*. *aegypti* from Africa, *Ae*. *albopictus* has become increasingly associated with urban and peri-urban landscapes as it has expanded its geographic range [5]. Within these landscapes, the species has become increasingly capable of exploiting human-made container habitat and human blood meal hosts.

**Fig 1:**
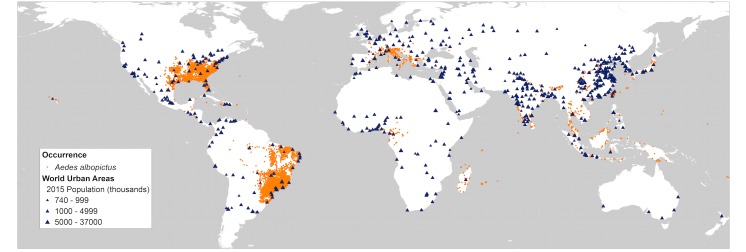
Global distribution of *Aedes albopictus* (orange dots) with superimposed major urban areas (blue triangles). *Ae*. *albopictus* occurrence data were from the database provided by [4]. Note in particular the extensive occurrence of cities in the United States within areas inhabited by this mosquito.

In recent years the introduction of *Aedes*-transmitted chikungunya and Zika arboviruses to the Western Hemisphere has raised important questions regarding the role that *Ae*. *albopictus* might play in arboviral transmission, especially in temperate regions where *Ae*. *aegypti* is rare but *Ae*. *albopictus* is increasingly abundant. Numerous lab studies indicate that *Ae*. *albopictus* can be equally competent (able to acquire and transmit pathogens) as *Ae*. *aegypti* for a suite of arboviruses, including chikungunya and Zika [6–11]. *Ae. albopictus* has also been associated with local arboviral transmission and disease outbreaks, specifically in temperate regions where *Ae. aegypti* is absent or uncommon[12–17]. However, *Ae*. *albopictus* is generally considered less important than *Ae*. *aegypti* for transmitting viral infections to humans because it has been shown to feed on a range of vertebrate species beyond human [18–20]. An *Ae*. *aegypti* mosquito that bites a human is highly likely to bite another human if it survives to feed more than once, making this species an important vector of arboviruses transmitted between humans [8,21–24]. *Ae aegypti* is also predominant in tropical regions where transmission cycles and viral amplification can be facilitated by longer seasons and greater opportunity for human-mosquito contacts. By contrast, *Ae*. *albopictus* has a far greater capacity than *Ae*. *aegypti* for exploiting a range of climates and habitat types, with established *Ae*. *albopictus* populations in rural and urban landscapes across both tropical and temperate regions [4,25] (Fig 1). Likewise, while *Ae*. *albopictus* host biting behavior is variable across its introduced range, urban regions can be focal areas of predominantly human biting [6,26–30]. In the United States, *Ae*. *albopictus* is now widespread throughout the eastern portion of the country, with increasingly urban association as the species has spread northward [5,31,32]. Increases in geographic range, urban occupation, and human biting, would all seem to intensify the potential for this vector to transmit arboviruses to humans. A quantitative evaluation is required to better understand how this behavioral plasticity and variable urban densities influence risk of local outbreaks of arboviral infection in temperate regions, including the densely populated eastern United States.

Many modeling efforts and risk predictions generate inference based on mean vector densities, human biting rates and other parameters that inform vectorial capacity. There are two limitations in this approach. First, data limitations mean that parameters are derived from data collected across very different landscapes in the species’ native and invasive range. Second, emergent outbreaks like the spreading Zika crisis and the more limited but still alarming human impacts of dengue emergence in Japan or chikungunya in Italy are not the outcome of average conditions–outbreaks occur when a suite of (often extreme or unusual) conditions align. Our goal in this paper is to quantitatively evaluate the potential for *Ae*. *albopictus* vectored transmission cycles and local disease outbreaks of emerging Zika and chikungunya viruses in temperate U.S. cities. We define probabilistic parameter distributions that represent mosquito densities, human host-use, and specific vector competencies reported in the literature and employ a mathematical model that explores the full range of observed parameter values to identify conditions that would facilitate local outbreaks in human population centers.

## Results

**Fig 2:**
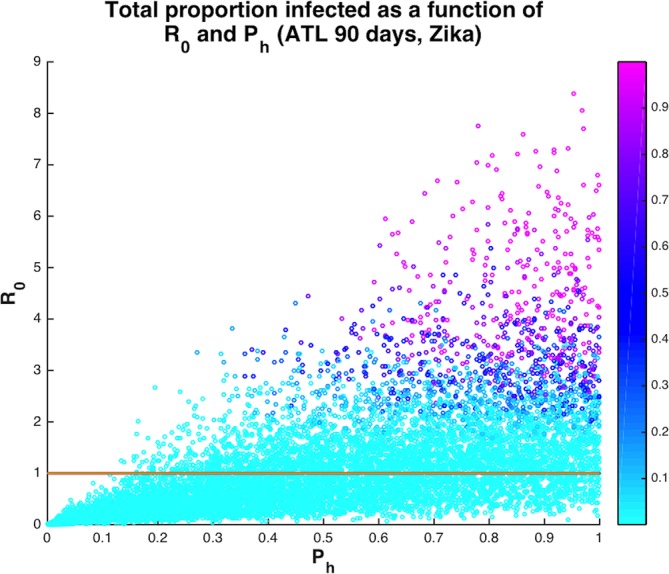
Proportion of the human population in a neighborhood infected with Zika virus at the end of the 90-day season in Atlanta (colorbar) as a function of R_0_ and P_h_, the proportion of blood meals that are human. The solid line is at R_0_ = 1. When P_h_> = 0.4, then 62.5% runs have R_0_>1 and 44.8% of runs result in at least 10 people infected after a single introduction. On the other hand, when P_h_ <0.4, then only 10.8% of runs have R_0_>1 and 4.0% of runs result in at least 10 people infected.

Our model draws on parameter values defined by field data and demonstrates how combinations of realistic parameter distributions can generate significant outbreak potential for chikungunya and Zika viruses in temperate U.S. cities (New York City, Philadelphia, Washington D.C., and Atlanta), where high *Ae*. *albopictus* densities are already reported. As expected, a majority of the model runs predicted that no outbreak would occur (R_0_<1). However, across the scenarios evaluated there is a persistent subset of runs where suites of realistic parameter combinations generate high R_0_ conditions that result in locally-transmitted human infections, including almost 45% leading to more than 10 new human infections if the mosquitoes bite humans even 40% of the time (Fig 2). For Zika virus, the average value of R_0_ across all 12 scenarios (encompassing 4 urban densities and 3 season lengths) was 1.1 with a median of 0.82 and a range of 0 to 13.1 (S1 Table). For chikungunya, the average value of R_0_ was 0.91 with a median of 0.68 and a range of 0 to 7.4 (S1 Table).

**Fig 3:**
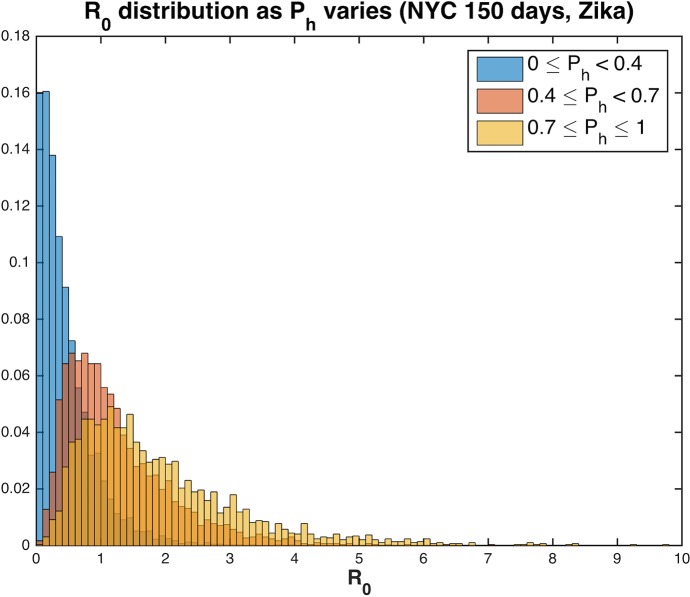
Distribution of R_0_ for Zika virus across ranges of human feeding rates, P_h_, for New York City. With P_h_≥0.4 probability of an outbreak increases significantly, resulting in 62.7% of runs with R_0_>1. However, when P_h_ < 0.4, the percent of runs with R_0_>1 decreases to 10.1% (for P_h_≥0.8, 76.3% of runs have R_0_>1). When P_h_ < 0.4, the mean value of R_0_ is 0.46, while for P_h_≥0.4, the mean value of R_0_ is 1.55 and if P_h_≥0.8, the mean value of R_0_ jumps to 1.97.

**Fig 4:**
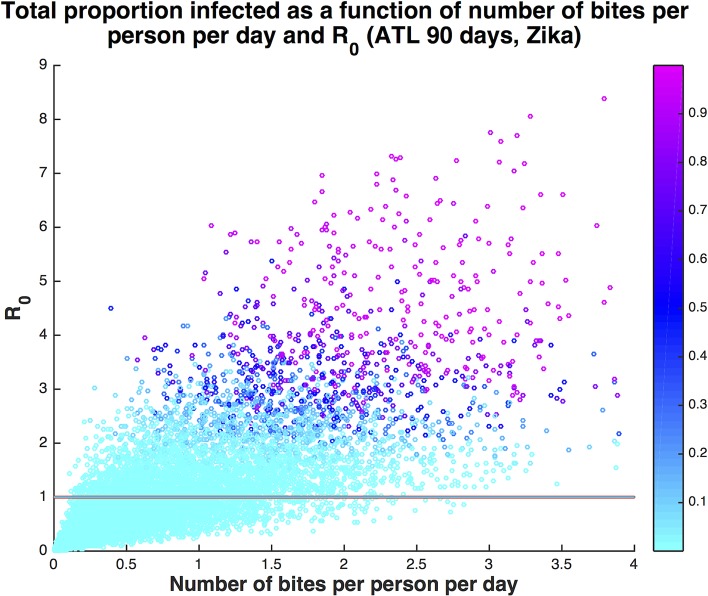
Proportion humans in a neighborhood infected with Zika virus (colorbar) as a function of number of bites per person per day and R_0_. The solid line is at R_0_ = 1. Even when the average number of bites per person per day is less than 1 (68% of all runs), many runs result in autochthonous transmission. Of the runs with number of bites less than 1, 34% result in at least one new infection, 10% result in at least 10 infections, and 2% result in at least 100 infections. If the season is extended to 120 days, that increases to 34%, 14%, and 4%, respectively.

We specifically evaluated how duration of active mosquito season following the arrival of an infectious traveler and propensity for biting diverse vertebrate species, where every non-human bite slows the transmission process, influence outbreak potential for different urban densities. As might be expected, higher probability of human host-use is associated with greater R_0_ (Fig 3). For a given seasonal duration and human population density, increasing the proportion of bites on humans in the mosquito population above 40% resulted in more model runs that returned R_0_ >1, signifying increased potential for local transmission and human disease even when a significant proportion of blood meals are from non-human animals (Fig 2). The average number of times a human was bitten per day in the model ranges from 0 to 4 bites. Even for number of bites per person per day below 1, there were several scenarios with significant onward transmission (Fig 4).

**Fig 5:**
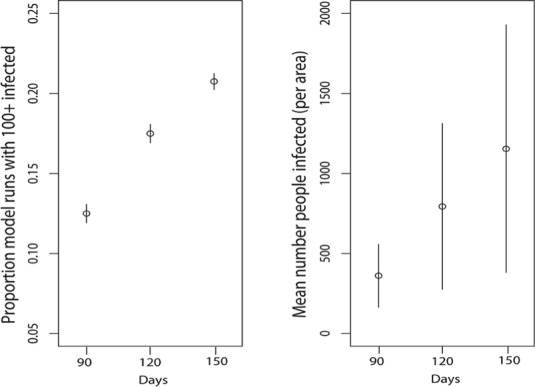
Season length (days, x axes) is positively associated with a) the proportion of model runs that resulted in 100 or more human infections with chikungunya virus and b) potential mean numbers of infected humans per square mile. Significant human infection is possible at even 90 days and uncertainty shown captures variability across cities, as well as human biting propensity and other parameter states. The mean number of people infected moves from 396 to 892 to 1376 and the median from 2.1 to 2.4 to 2.5 as season increases from 90 to 150 days.

Potential human infection was positively associated with seasonal duration representing the length of time with active, high-density mosquito populations following the introduction of an infectious traveler. For example, the 90-day scenario for Zika in Philadelphia resulted in 51.8% of runs with at least one new human infection from a single primary introduction and 14.4% resulted in more than 100 people infected. Across all scenarios, the 90-day season results in 14.4% of runs with greater than 100 people infected, 120-day season in 20.4% of runs with greater than 100 people infected and 150-day season in 24.8% of runs with greater than 100 people infected (S1 Table, Fig 5). So, while on average there is only one new infection generated following a single primary introduction during a season, the chance of a relatively large outbreak increases substantially with season length as more mosquitoes become infected. Extending the season also reduced the value of P_h_ needed to result in potentially severe outbreaks (S1 Fig). Although season length varies from year to year in the cities we investigated, the climate in Atlanta and Washington D.C. tends to support longer mosquito seasons of at least 4–5 months, corresponding to our 120 and 150-day seasons. On the other hand, New York City and Philadelphia would be more likely to have 90-day peak mosquito seasons, on average. However, anomalously warm (or cold) years could change mosquito season length and thus, risk.

**Fig 6:**
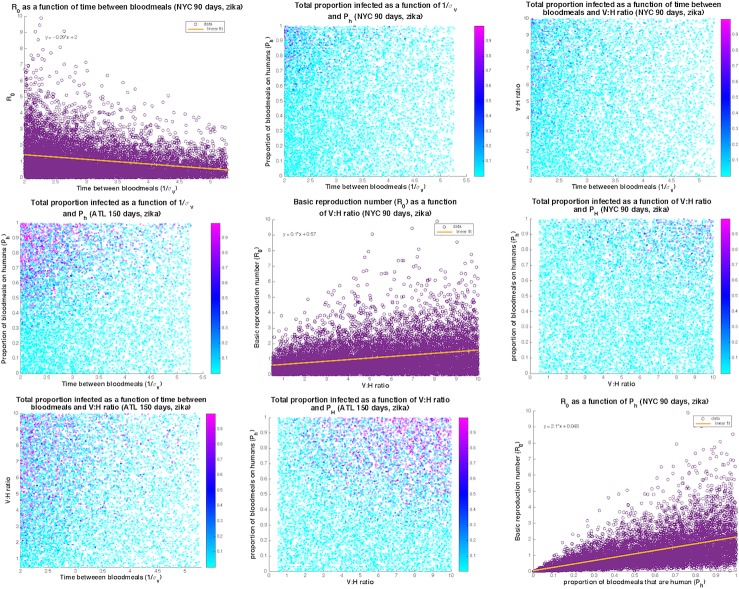
Plots of the top three most sensitive parameters for Zika as they relate to each other and to the percent of the population infected by the end of the outbreak for New York City (upper right triangle) and Atlanta (lower left triangle) and to R_0_ (diagonal). The range of parameters resulting in large absolute numbers infected is larger for longer mosquito seasons (Atlanta). Large outbreaks tend to be restricted to high biting rates (time between bloodmeals) and high proportions of bites on humans.

To quantify sensitivity of output to specific parameter combinations and inform targets for surveillance and mitigation, partial rank correlation coefficients were calculated separately for Zika and chikungunya. Values of R_0_ for Zika were most sensitive to variation in the percent of bites on humans, initial mosquito density, and mosquito biting frequency (Table 1). Chikungunya’s R_0_ was also highly sensitive to percent of bites on humans versus dead-end hosts and had similar sensitivies to the other parameters as Zika. For New York City, with the shorter 90-day mosquito season, large outbreaks are generally characterized by a vector to host ratio larger than 2, time between bloodmeals less than 4.5 days and proportion of bites on humans greater than 0.5 (upper right triangle of Fig 6). If one of these parameters is on the high end of its range, then the other two can be in mid-range and still result in a large outbreak. As the mosquito season lengthens, the range over which large absolute numbers of people may be infected increases. For Atlanta with a 150-day peak mosquito season, large outbreaks are occurring with vector to host ratios as low as 1, time between bloodmeals as high as 5 days and proportion of bites on humans as low as 0.3 (lower left triangle of Fig 6). The highest R_0_ values are seen for frequent biting (less than 3 days between bloodmeals) and high proportion of bites on humans (diagonal, Fig 6).

**Table 1:** Sensitivity Analysis. PRCC values computed for R_0_ and proportion of the human population infected at the end of season for Zika and chikungunya (Philadelphia, 90-day season). Absolute values close to zero indicate low sensitivity and absolute values close to one indicate high sensitivity. Negative values indicate an inverse relationship between the parameter and the quantity of interest (output).

Parameter	nu_v	mu_v	beta_hv	K_v	sigma_v	sigma_h	nu_h	beta_vh	gamma_h	P_h
PRCC value R_0_ ZIKA	0.1575	-0.5235	0.5668	0.6132	0.5514	0.2267	-0.0061	0.559	-0.4739	0.8871
PRCC value proportion infected ZIKA	0.2027	-0.4182	0.5804	0.6263	0.5669	0.2382	0.0269	0.5716	-0.4066	0.8918
PRCC value R_0_ CHIK	0.1158	-0.4793	0.6956	0.6148	0.5469	0.215	-0.0034	0.3039	-0.281	0.8847
PRCC value proportion infected CHIK	0.148	-0.3891	0.702	0.6227	0.5541	0.2203	0.008	0.3115	-0.2543	0.8873

While variable human density across the representative cities does not influence the mean R_0_ values or percent of runs with more than 100 human infections, the absolute size of the outbreaks and mean percent of the population infected are associated with human density. For very low human density regions, outbreaks with more than 100 infections would become highly improbable or even impossible, but these results hold for human densities seen in urban areas. For example, the mean number of people infected for a 90-day season in Atlanta (lowest human density) is 175, while for New York (highest human density) it is 676 (S1 Table). Note that we are considering local transmission within a square mile plot, so the percent infected is the percent of people living in or spending significant time in that local area (Table S4 in the S1 Text gives number of people per square mile).

## Discussion

Our model indicates that risk of local transmission of Zika and chikungunya viruses and human disease outbreaks in temperate U.S. cities is considerable. Regardless of season length, there is a greater than 50% chance of some onward transmission if a human case is introduced to a temperate, urban landscape with high *Ae*. *albopictus* population density. This means that one of every two infectious travelers could initiate local transmission under the right conditions. This is, of course, not a prediction that we expect to be validated by documented human outbreaks. For one thing, both viruses can be predominantly asymptomatic in humans. But perhaps more importantly, the suite of parameters that is necessary to achieve this R_0_ represents a pretty specific chain of events and conditions and while the parameter values are each realistic, we actually don’t have the data to assess how frequently the suite of conditions occur, in real space, that support mosquito exposure to an infectious traveler, extrinsic incubation and then subsequent transmission to a second human host. The first necessary condition is high population abundance of *Ae*. *albopictus*. Studies confirm high densities and growing populations of this species across the eastern U.S. and as far north as New York [31–33]. A second necessary condition is that the female *Ae*. *albopictus* must bite humans at least 40% of the time. The Asian tiger mosquito’s vectorial capacity is persistently questioned because the propensity for biting humans versus other vertebrates varies widely, as the species appears to opportunistically bite the most available vertebrates [19,20,26,28–30,34–37]. We show that while a higher probability of human host-use is associated with greater R_0_, increasing the proportion of bites from humans above 40% increased potential for local transmission and resulting human disease. This percentage threshold of human biting is frequently exceeded in studies within urban landscapes [26,28–30,37]. A third condition that our model confirms is the importance of seasonal duration. When mosquito density and biting activity remains high for a longer period of time there is greater potential for local transmission. This duration is influenced by seasonal temperatures as well as the timing of when the first infectious traveler is accessible to mosquito bites.

The ability to manage mosquito population growth and associated arboviral transmission to humans requires early recognition of conditions that facilitate high vector population density and human biting behavior. When these conditions are favorable, transmission following the arrival of an infectious traveler can progress rapidly, as demonstrated in the 2014 urban dengue outbreak vectored by *Ae*. *albopictus* in Tokyo, Japan [38,39]. Although some researchers consider non-zoonotic arboviruses (e.g., Zika, chikungunya, and dengue viruses) unlikely to become endemic in temperate regions where seasonality is a strong filter on transmission, we demonstrate that a conditional series of non-average events can result in local pathogen transmission and outbreaks of disease in humans. This study confirms that non-average conditions likely to facilitate transmission after the introduction of an infectious traveler include years with particularly long, warm seasons in regions with high densities of competent vectors, high biting rates, and higher proportions of bites on humans, which often corresponds to high human density (Fig 6).

**Fig 7:**
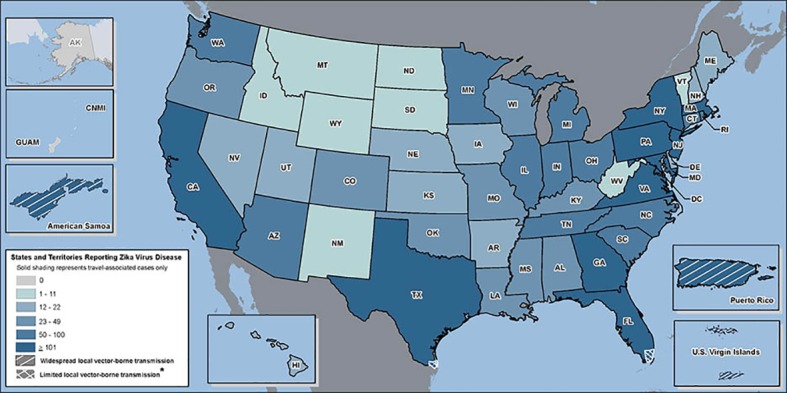
A map of 2016 introductions of Zika virus to the United States (CDC Zika website, http://www.cdc.gov/zika/geo/united-states.html, accessed January 3, 2017). As of January 1, 2017 there were 4,592 travel-associated Zika cases and 216 locally acquired Zika cases reported in the United States in 2015–2016. In 2015, a total of 896 travel-related chikungunya cases and 1 locally acquired case were reported in the United States.

Recent introductions of both chikungunya virus and Zika virus to the Western Hemisphere have been followed by rapid intensification of human disease and/or broad geographical spread, particularly in and near urban centers [40,41]. Public health officials need validated assessments of how likely these viruses will be locally transmitted, even in temperate regions where *Ae*. *albopictus* populations are abundant and introduction of an infected traveler is likely. There has been repeated documentation of return and visiting travelers infected with chikungunya and more recently, Zika, over the past four years (Fig 7). For example, in the first six months of 2016 alone, 182 (5%) of 3605 residents of New York City who had returned from an area with ongoing Zika virus transmission were infected with Zika virus, as confirmed by RT-PCR or serologic testing [42]. These travelers can serve as sources of local transmission particularly if they are asymptomatic.

The R_0_ models quantify the probability of at least one local transmission event for each infected individual entering one of the cities at the beginning of the transmission season. Arrivals later in the season would lead to lower outbreak sizes, but multiple infected individuals arriving at once would increase the probability of a larger outbreak. While local chikungunya transmission has not yet led to significant human disease in the contiguous United States, our results suggest that the chance for local Zika transmission is greater. Our predictions show that the risk of local transmission and human infection with Zika is, on average, slightly higher than for chikungunya virus in temperate cities, which is consistent with differences in human infection rates reported on Yap island (73% human population with Zika infection [43]) versus chikungunya prevalence on Reunion Island (35% population prevalence[44]). Number, timing, and location of introductions will affect probability of successful introduction as well. In 2015, there were 38 chikungunya introductions to the state of New York, 3 introductions to Pennsylvania, 0 to Washington D.C., 9 to Maryland, and 4 to Georgia from June to August (http://diseasemaps.usgs.gov/mapviewer/). Greater certainty in specific parameter values, particularly vector competence of *Ae*. *albopictus* for Zika transmission, will increase the precision of our model predictions.

We chose to focus on urban areas dominated by *Ae*. *albopictus*, which has a much larger distribution in the temperate zone worldwide than does *Ae*. *aegypti*. Consequently, we did not include Gulf States such as Florida, Louisiana, and Texas in our analysis. High-density urban areas in these states are often dominated by *Ae*. *aegypti* or a mixture of *Ae*. *aegypti* and *Ae*. *albopictus*. For example, Miami’s urban regions (and the Florida Keys), where local transmission of dengue, chikungunya, and Zika have taken place, have high densities of *Ae*. *aegypti* [45–49]. Although published data are sparse for Houston, where local dengue transmission has occurred, there is evidence for similar mosquito distribution patterns there [50]. Adapting the model to include *Ae*. *aegypti* and mixtures of *Ae*. *aegypti* and *Ae*. *albopictus* would be an important next step. Based on our model results, we predict that the much longer peak mosquito seasons in the Gulf States along with higher mosquito feeding rates and very high proportion of bites on humans by *Ae*. *aegypti* would result in even higher risk of arboviral outbreaks (see, e.g. Fig 6).

As with any modeling effort, the results presented are contingent on the assumptions made in defining structure and parameterization. Our model assumes that all parameters are independent. However, it is likely that some are correlated; for instance temperature may simultaneously influence vector competence, biting rate, and vector life history [51,52]. To our knowledge, there have been no lab or field studies examining the effects of temperature on Zika replication in mosquitoes and there is very little on the affect of temperature on chikungunya’s EIP [9,52,53]. Although for dengue, increased temperature tends to decrease the EIP up to a point [54,55], increased temperature also tends to decrease mosquito lifespan, leading to nonlinear and complex relationships between temperature and transmission rates[9]. Moreover, most studies have been done in the lab and at constant temperatures for mosquito populations from one location. How this translates to field conditions and different mosquito populations is unclear since there is evidence for mosquito adaptation to local climate [9] and different dengue EIP responses to daily temperature fluctuations [51,54]. More data, including relationships between temperature regimes common to temperate regions and EIP and mosquito lifespan, are needed to better understand co-variation in mosquito and pathogen dynamics in real field conditions.

Likewise, current studies demonstrate considerable variation in *Ae*. *albopictus* density and human biting within a city and across land-use types [21,39,56]. More field data and behavioral evaluation are needed to refine model assumptions and parameters regarding when and where mosquito density and percent human feeding is likely to facilitate onward human transmission. For dengue and *Ae*. *aegypti*, mosquito density and virus transmission are closely related, but at different spatial scales [57], with the scale to best consider dengue transmission still unclear. Although there has been some success correlating vector abundance or environmental factors with risk for dengue[58], other studies have shown poor correlation [59], particularly for urban areas with mosquitoes that breed in human-created habitat and thus depend less on environmental factors such as rainfall[60]. Percent human feeding and mosquito density data are needed to rigorously assess the thresholds and scales at which mitigations of mosquito abundance and human biting rates might be effective.

The model assumes that mosquito population density (and vector:human ratio) are at the carrying capacity (and vector:human ratio) used to initialize the model throughout the specified seasonal duration (i.e., 90, 120 or 150 days). This density level defines the vector to human ratio and strongly influences R_0_ and numbers of additional human infections within a season. Our parameters represent ranges that capture real observed densities, but due to limitations in available field data, do not necessarily represent the relative duration of how long peak densities are generally maintained. Ongoing work in Maryland supports the assumption that a 90 day season of high *Ae*. *albopictus* density is likely in most years in that region [32]. It takes mosquito populations several weeks to ramp up to high densities. The beginning of our season is assumed to be when mosquitoes are at the high densities that can persist throughout the summer and early fall across the range of cities included. Likewise, the model does not incorporate mitigations or behavior changes, so it represents *potential* outbreak size rather than probable outbreak size since once autochthonous transmission is detected, significant mitigation efforts are likely. However, it should be noted that because 80% of Zika infections are asymptomatic[43], time to detection of an outbreak and response could be longer than for other diseases. Chikungunya, on the other hand, is highly symptomatic (around 80–90% of those infected exhibit symptoms, [61,62]), so it is more likely to be detected and motivate mitigation efforts.

Finally, the model assumes that within the square-mile area considered, mosquitoes and humans are homogeneous in space and can be characterized by mean densities. The scale is consistent with CDC’s assessment of risk in Miami (one sq mile around area with known transmission, https://www.cdc.gov/zika/intheus/florida-update.html). However, both mosquito and human densities can vary across space, resulting in varying risk of human-mosquito contact. Depending on human movement, daily activities, and vector exposure (which will depend upon socioeconomic factors, among other things), our model may under- or over-estimate risk, as has been illustrated by data and in individual-based models for mosquito-borne disease that incorporate more heterogeneity [63–66]. However, models that assume even mixing have performed relatively well and been useful in understanding risk of urban mosquito-borne disease transmission [67–69].

Scientists and public health officials involved with arbovirus transmission have had limited ability to make credible predictions, in part based on limited information about conditions that permit an outbreak and the likelihood those conditions will be met. Our model provides quantitative assessments of the probability of an outbreak (R_0_) and the potential numbers of human victims when key parameter values can be specified. Guided by published data on virus and mosquito vital rates, the model indicates that outbreaks can plausibly occur in major cities in the eastern United States, with hundreds of potential victims in localized areas, under conditions that are not atypical. The model suggests that outbreaks are more likely in urban areas with higher human and mosquito population densities, in years and cities with longer growing seasons, when infected travelers arrive early in the growing season, and when *Ae*. *albopictus* have fewer non-human hosts that result in wasted bites. These conditions are most likely met in urban landscapes where social, structural and environmental inequities facilitate human-mosquito contact and potentially limit early detection and mitigation of local transmission. Climate change, urban wildlife ecology, and human behavior all would appear to strongly influence the probability of new outbreaks in major U.S. cities.

## Methods

### Ethics Statement

The published literature we used does not reveal confidential information regarding human participants, so this study did not require IRB approval and no confidential human data has been revealed here.

**Fig 8:**
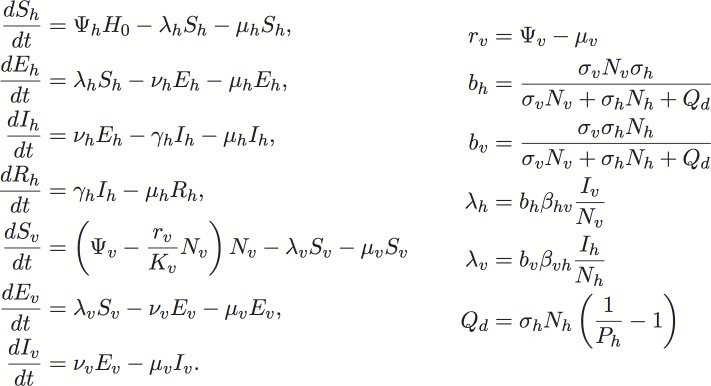
Model equations. The model follows standard epidemiological model structure and assumes that all humans are susceptible (S_h_), exposed and incubating (E_h_), infectious (I_h_), or recovered and immune (R_h_). Likewise, mosquitoes are assumed to be susceptible (S_h_), exposed and incubating (E_h_), or infectious (I_h_). The force of infection terms, λ_v_ and λ_h_, depend on total number of mosquitoes, N_v_, and total number of humans, N_h_, along with the mosquito biting rates, σ_v_, human bite availability, σ_h_, and bites on alternate hosts, Q_d_. All parameter definitions and values can be found in the Supplementary Text.

We used a compartmental mathematical transmission model adapted from [70] to evaluate the potential for *Ae*. *albopictus* transmission of Zika and chikungunya virus to humans following the introduction of an infectious traveler. The model follows standard epidemiological model structure and assumes that all humans are susceptible (S_h_), exposed and incubating (E_h_), infectious (I_h_), or recovered and immune (R_h_). Likewise, mosquitoes are also assumed to be susceptible (S_h_), exposed and incubating (E_h_), or infectious (I_h_). The model includes population dynamics for mosquitoes with density-dependent emergence of adult female mosquitoes and a carrying capacity, K_v_. We adapted the Manore et al. 2014 model to sample from literature-informed variation in parameter space, and account for variability in use of human blood meal hosts (see Fig 8 for equations and parameter definitions). Studies demonstrate that propensity for human biting by *Ae*. *albopictus* across its invasive range varies widely and that the species appears to opportunistically bite whatever birds or mammals most readily available [18,20,26,28,30,34,35,71], although some studies indicate a human preference [37]. We assumed that of the total number of mosquito bites per day a certain proportion, P_h_, are on humans and 1- P_h_ are on alternate hosts. We assumed that the non-human alternate hosts are not susceptible to the pathogen and thus, when an infected mosquito bites a non-human animal, the bite is “wasted” in the sense that the virus is not passed on to the animal. However, if the infected mosquito survives to bite again and the next bite is on a susceptible human, then the infected mosquito could pass on the virus to the human. The model does not consider other modes of transmission such as male to female sexual transmission of Zika in humans.

A number of epidemiological models have considered arboviral transmissions (particularly dengue and chikungunya) focusing on different aspects of disease transmission [67,70,72–76] and characteristics such as seasonality, temperature dependence, cross-immunity with multiple strains, and control measures [77–85]. Several recently published modeling studies for Zika transmission have focused on fitting models to current transmission in the South Pacific and South and Central America [68,86–88] with *Ae*. *aegypti* dominating transmission. Only one, to our knowledge, has considered risk in temperate regions of Europe with transmission by *Ae*. *albopictus* [89]. Our model assesses risk in temperate regions of the United States dominated by *Ae*. *albopictus* and with no current local transmission of Zika or chikungunya, but with frequent introductions from other regions. We explicitly include human and mosquito dynamics and determine risk by computing both R_0_ and absolute size of a potential outbreak.

We used parameter values for chikungunya from [70] with an updated baseline and range for chikungunya’s extrinsic incubation period (EIP) based on meta-analysis in [91]. Zika parameters for human incubation and infectious periods (ranges are wide due to uncertainty, 3–12 days and 3–14 days, respectively), transmission probabilities given an infected contact (again, ranges slightly larger than for chikungunya based on the few current models and high uncertainty, 0.1–0.75 for Zika as opposed to 0–0.54 for chikungunya), and the EIP (higher than chikungunya), were based on the most up-to-date Zika field and modeling literature (see the S1 Text and S2 Table for references and data used).

In the model, mosquitoes bite infected or susceptible humans at a rate defined by the per-human vector density and the propensity for biting humans versus other animals. Mosquitoes become infectious and transmit virus to susceptible humans as a function of this biting rate, the number of infected humans, and vector competence (S1 Text). Vector competence integrates mosquito survival and EIP for the specific virus along with transmission probability given a bite on a susceptible human. Parameter values informing *Ae*. *albopictus* life history and specific vector competence for Zika and chikungunya virus transmission were estimated from published studies (S1 Text and S2 Table). To inform parameters related to *Ae*. *albopictus* population dynamics and vector competence, separate searches for *Aedes albopictus* survival, death, emergence and egg-laying rates and for Zika and chikungunya and *Ae*. *albopictus* were performed to supplement the studies and parameter values used in [70]. Details of the data and studies chosen are in included in the S1 Text description of the model.

Vector densities were varied from 0.5 to 10 times the human density in a square mile (2.59 square kilometers). Vector density was assumed to be at carrying capacity, K_v_, for the duration of the season-length specified (90 to 150 days). We are thus assuming that fluctuations in vector abundance are minor within the time frame of our predictions and that those fluctuations remain within our vector-to-host ratio range. We also used human biting rate experiments to verify that the ratio we used resulted in a realistic number of bites per person per day (see model description in S1 Text and S2 Table for a more detailed outline of our rationale). Carrying capacity was drawn randomly from a uniform distribution bounded by values representing 0.5 to 10 mosquitoes per human host. A uniform distribution was chosen due to lack of more informative data. We considered representative human density per square mile representing four eastern U.S. cities with high to low urban residential densities: New York City (NY), Philadelphia (PA), Washington (DC), and Atlanta (GA). The vector density range captures large variability in published (S1 Text and S2 Table, mean vector-to-host ratios ranging from 3.1 to 10) and current data on *Ae*. *albopictus* populations in urban regions [25].

We varied the peak mosquito season lengths from 90 to 150 days to capture the effect of season length on risk. The short, 90-day season could represent either a later seasonal introduction of an infectious traveler or a shorter northeastern season (i.e., June-August), while a 150-day season represents a potential mid-May to mid-October season with early viral introductions. In general, Atlanta and Washington D.C./Baltimore would be expected to have longer mosquito seasons corresponding to the 150-day season in the model and New York and Philadelphia would be expected to have shorter mosquito seasons corresponding to the 90-day season in the model. In addition to human and vector density, percent of human blood meals, and season length, we varied human and mosquito incubation periods, mosquito biting rate, human biting tolerance, human infectious period, and transmission probabilities given an infected contact, across ranges based on the literature (see S1 and S2 Tables and the S1 Text for parameter and variable definitions and parameter values).

The quantities of interest computed from the model were the basic reproduction number (R_0_) and the cumulative absolute number of people infected at the end of the season given an introduction at the beginning of the season. The basic reproduction number is the expected number of secondary cases from one introduced case in a fully susceptible population. We used the next generation method to compute the basic reproduction number [90], which in that framework is the geometric mean of the expected number of transmissions to mosquitoes from one infected human and the expected number of transmissions to humans from one infected mosquito in fully susceptible populations (S1 Text). The cumulative number of people infected was computed by running numerical simulations of the model in MATLAB for the given seasonal duration. The model was run for local transmission in a square mile using each city’s specific human population density. The model was initialized with mosquitoes at carrying capacity and fully susceptible and one infected human introduced on day 1.

In order to fully explore the variation in parameter values and risk, we sampled from the given parameter ranges (S1 Text) and computed our quantities of interest using 10,000 randomly selected parameter combinations for each of the four human densities and three seasonal duration scenarios. We varied all but the two least sensitive parameters of the model (human death rate and mosquito emergence rate, which does not affect mosquito densities once the population is at carrying capacity). The model’s ability to generate realistic values for bites per human per day and other derived statistics was confirmed. Model validation was done previously using baseline parameters for chikungunya and dengue and compared favorably to observed outbreaks [70]. We did not have access to data to validate the model’s ability to predict observed Zika case data where *Ae*. *albopictus* transmission has been confirmed.

## Supporting Information

S1 Fig Proportion of the human population infected with Zika virus in New York City as a function of P_h_ (proportion of blood meals on humans) and season length. From left to right, 90-day, 120-day, and 150-day peak mosquito seasons are shown. As season length increases, the percent of serious outbreaks increases and the needed percent of human feeding to result in a serious outbreak decreases.
(TIFF)

S2 Fig Proportion of the human population infected with chikungunya at the end of the 90-day season in Atlanta as a function of R_0_ and P_h_ (proportion of blood meals that are human). The red line is at R_0_ = 1.
(TIFF)

S3 Fig Distribution of chikungunya R_0_ across ranges of human feeding rates, P_h_, for New York City. (TIFF)

S4 Fig Proportion humans infected with chikungunya as a function of number of bites per person per day and R_0_. The solid line is at R_0_ = 1.
(TIFF)

S5 Fig Proportion of the population infected with chikungunya in New York City as a function of P_h_ (proportion of blood meals on humans) and season length. From left to right, 90-day, 120-day, and 150-day peak mosquito seasons are shown.
(TIFF)

S1 Table Summary results for our quantities of interest, R_0_ and total number of people infected, for each scenario (city, season length, virus). (XLSX)

S2 Table Summary of references and data used to determine parameter values. (XLSX)

S1 Text Detailed description of the model and parameter values used for simulations. (PDF)
