# Supplementary figures and images for: Defining the Risk of Zika and Chikungunya Virus Transmission in Human Population Centers of the Eastern United States
Source: PLoS Negl Trop Dis. 2017 Jan 17;11(1):e0005255. doi: 10.1371/journal.pntd.0005255 (PMC5319773; doi:10.1371/journal.pntd.0005255)

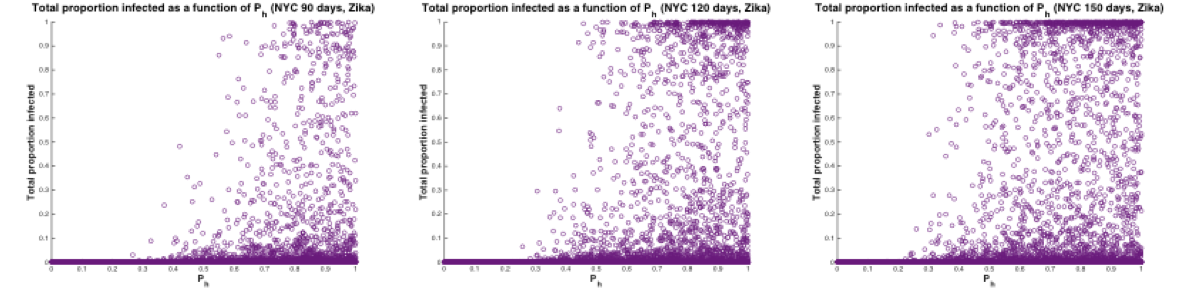

Supplement: S1 Fig — From left to right, 90-day, 120-day, and 150-day peak mosquito seasons are shown. As season length increases, the percent of serious outbreaks increases and the needed percent of human feeding to result in a serious outbreak decreases. (TIFF) [file pntd.0005255.s001.tiff]

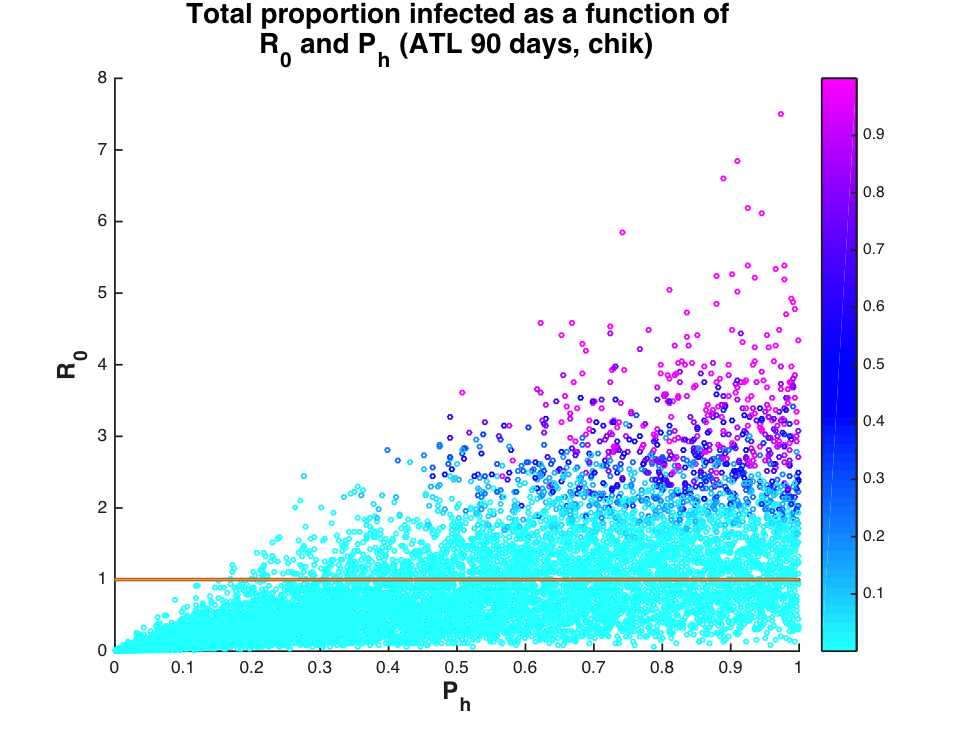

Supplement: S2 Fig — The red line is at R0 = 1. (TIFF) [file pntd.0005255.s002.tiff]

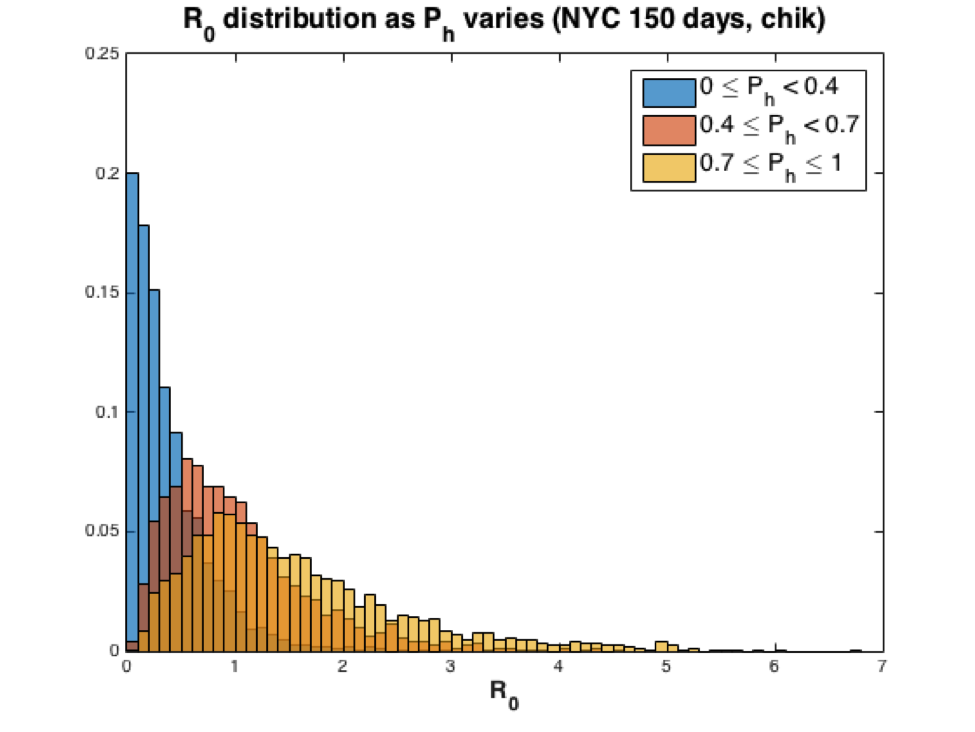

Supplement: S3 Fig — (TIFF) [file pntd.0005255.s003.tiff]

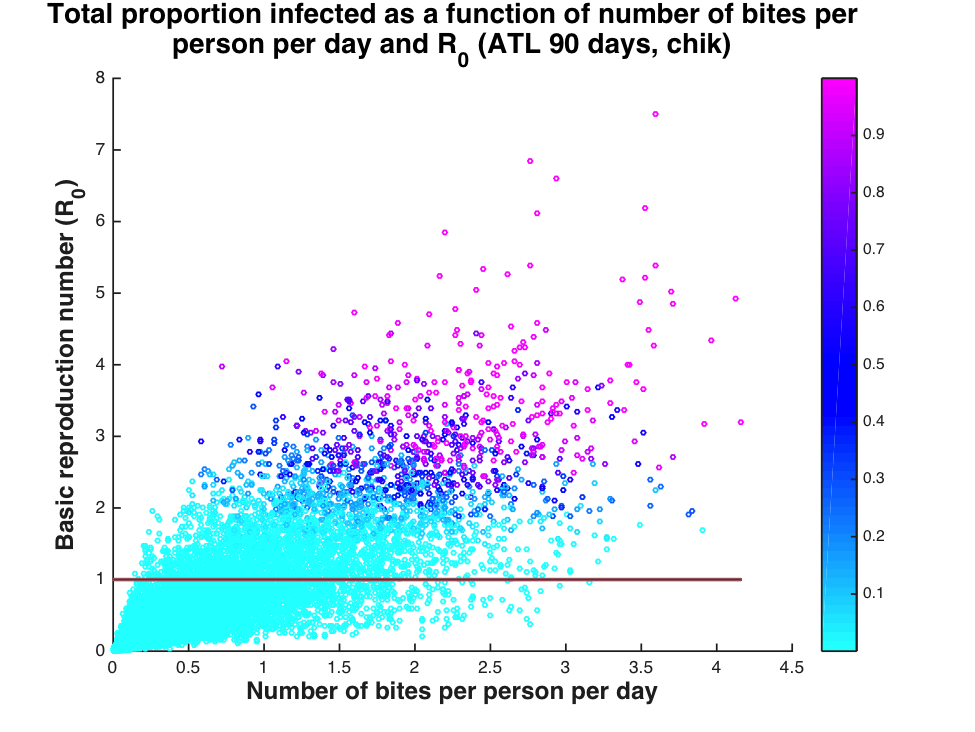

Supplement: S4 Fig — The solid line is at R0 = 1. (TIFF) [file pntd.0005255.s004.tiff]

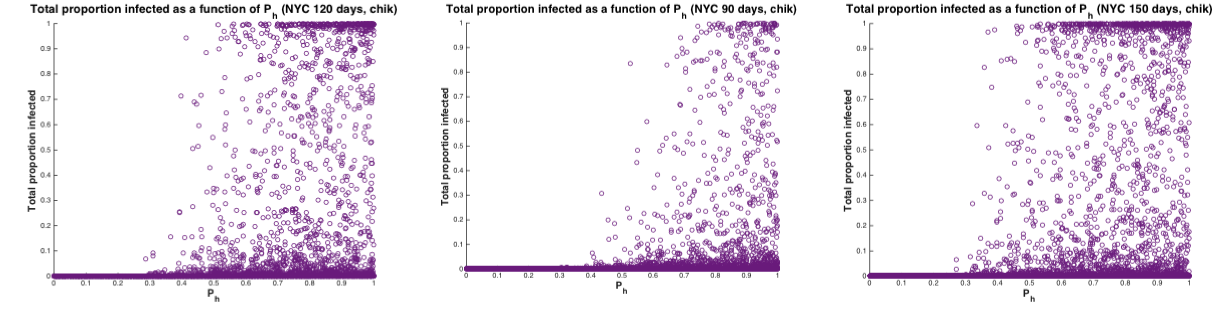

Supplement: S5 Fig — From left to right, 90-day, 120-day, and 150-day peak mosquito seasons are shown. (TIFF) [file pntd.0005255.s005.tiff]
