# Supplementary material for: Defining the Risk of Zika and Chikungunya Virus Transmission in Human Population Centers of the Eastern United States
Source: PLoS Negl Trop Dis. 2017 Jan 17;11(1):e0005255. doi: 10.1371/journal.pntd.0005255 (PMC5319773; doi:10.1371/journal.pntd.0005255)
Supplement: S1 Text — (PDF) [file pntd.0005255.s008.pdf]

Supplementary Material S8: Model and Parameter Descriptions

The state variables (Table S2) and parameters (Table S3) for the model were derived from [1] and satisfy the equations

$$\frac{dS_h}{dt} = \Psi_h H_0 - \lambda_h(t) S_h - \mu_h S_h, \tag{0.1a}$$
$$\frac{dE_h}{dt} = \lambda_h(t) S_h - \nu_h E_h - \mu_h E_h, \tag{0.1b}$$
$$\frac{dI_h}{dt} = \nu_h E_h - \gamma_h I_h - \mu_h I_h, \tag{0.1c}$$
$$\frac{dR_h}{dt} = \gamma_h I_h - \mu_h R_h, \tag{0.1d}$$
$$\frac{dS_v}{dt} = h_v(N_v) N_v - \lambda_v(t) S_v - \mu_v S_v \tag{0.1e}$$
$$\frac{dE_v}{dt} = \lambda_v(t) S_v - \nu_v E_v - \mu_v E_v, \tag{0.1f}$$
$$\frac{dI_v}{dt} = \nu_v E_v - \mu_v I_v. \tag{0.1g}$$

The human population is divided into susceptible (S_h), exposed/incubating (E_h), infectious (I_h), and recovered/immune (R_h) compartments. The female mosquito population is divided into susceptible (S_v), exposed/incubating (E_v), and infectious (I_v) compartments. The total population sizes are $N_h = S_h + E_h + I_h + R_h$ and $N_v = S_v + E_v + I_v$ for humans and mosquitoes, respectively. The mosquito birth rate is

$$h_v(N_v) = \Psi_v - \frac{r_v}{K_v} N_v,$$

where Ψ_v is the natural birth rate in the absence of density dependence, $r_v = \Psi_v - \mu_v$ is the intrinsic growth rate of mosquitoes in the absence of density dependence, and K_v is the carrying capacity of the female mosquitoes. Then,

$$\frac{dN_v}{dt} = \left(\Psi_v - \frac{r_v}{K_v} N_v\right) N_v - \mu_v N_v = r_v \left(1 - \frac{N_v}{K_v}\right) N_v$$

and the positive mosquito population equilibrium is K_v.

We extended the biting rate in [1] to include an alternate host species, properly apportioning the total number of mosquito bites among hosts (using methods similar to [2]) so that only a proportion, P_h, of mosquito bites per day are on humans. Following the human-mosquito contact formulation in [3, 1], σ_v is the maximum rate at which a mosquito will seek a blood-meal, and σ_h (σ_d) is the maximum number of bites that a human (alternate dead-end host) can support per unit time. Then, $\sigma_v N_v$ is the maximum number of bites the mosquito population seeks per unit time and $\sigma_h N_h + \sigma_d N_d$ is the maximum number of host bites available per unit time. Since alternate hosts for *Aedes albopictus* can vary, we will group $\sigma_d N_d$ into one parameter, $Q_d = \sigma_d N_d$ that represents biting pressure on alternate hosts in general. The total number of mosquito-host contacts is then

$$b = \frac{\sigma_v N_v (\sigma_h N_h + Q_d)}{\sigma_v N_v + \sigma_h N_h + Q_d} \tag{0.2}$$

which depends on the population densities of humans, alternate hosts, and mosquitoes. The advantage of using this biting rate, as opposed to the more standard frequency-dependent contact

rates, is that it can handle the whole range of possible vector-to-host ratios, whereas frequency or density-dependent contact rates have limited ranges of vector-to-host ratios across which they are applicable [4]. We define

$$b_h = \frac{b}{N_h} \cdot \frac{\sigma_h N_h}{\sigma_h N_h + Q_d} = \frac{\sigma_v N_v \sigma_h}{\sigma_v N_v + \sigma_h N_h + Q_d} \tag{0.3}$$

as the number of bites per human per unit time, and

$$b_v = \frac{b}{N_v} \cdot \frac{\sigma_h N_h}{\sigma_h N_h + Q_d} = \frac{\sigma_v \sigma_h N_h}{\sigma_v N_v + \sigma_h N_h + Q_d} \tag{0.4}$$

as the number of bites per mosquito per unit time on a human. Then, the forces of infection are

$$\lambda_h = b_h \beta_{hv} \frac{I_v}{N_v}, \tag{0.5}$$

$$\lambda_v = b_v \beta_{vh} \frac{I_h}{N_h}. \tag{0.6}$$

The fraction of bites on humans is

$$P_h = \frac{\sigma_h N_h}{\sigma_h N_h + Q_d}. \tag{0.7}$$

Given a known fraction of blood meals on humans, P_h, the total available bites on alternate hosts is solved as

$$Q_d = \sigma_h N_h \left(\frac{1}{P_h} - 1 \right). \tag{0.8}$$

The basic reproduction number for this model is the geometric mean of R_{hv} and R_{vh}. We defined R_{hv} as the expected number of secondary human cases resulting from one introduced infected mosquito in a fully susceptible population and R_{vh} as the expected number of secondary mosquito cases resulting from one introduced infected person in a fully susceptible population. So, $R_0 = \sqrt{R_{hv} R_{vh}}$ where

$$R_{hv} = \frac{\nu_v}{\mu_v + \nu_v} \frac{H_0 M}{\mu_v} \beta_{hv} \tag{0.9}$$

$$R_{vh} = \frac{\nu_h}{\gamma_h + \mu_h} \frac{K_v M}{\nu_h + \mu_h} \beta_{vh} \tag{0.10}$$

where

$$M = \frac{\sigma_v \sigma_h}{\sigma_v K_v + \sigma_h H_0 + Q_d}. \tag{0.11}$$

The first terms of R_{hv} and R_{vh} are the probability of surviving the incubation period (non-trivial for mosquitoes). The second terms are the average number of bites on humans an infected mosquito will make while infectious and the average number of mosquito bites a human will get while infectious, respectively. The final terms are probability of successful transmission given an infectious contact.

The EIP (extrinsic incubation period) is the time it takes for a mosquito to become infectious after exposure via a viremic bloodmeal. The average EIP for chikungunya in *Ae. albopictus* most likely ranges between 5.9 and 8.2 days based on a recent meta-analysis of lab and field studies (Christofferson at el. 2014 [28] and references therein). We computed the EIP of Zika virus by fitting a cumulative exponential distribution to the data in [15] and the resulting value was supported by [29, 30], who found that the EIP was most likely > 7 days and between 9 and 11 days. However, those studies did not provide the necessary data to use explicitly in our computation of the EIP.

Table S2: State variables for the model (0.1).

S_h:	Number of susceptible humans
E_h:	Number of exposed humans
I_h:	Number of infectious humans
R_h:	Number of recovered humans
S_v:	Number of susceptible mosquitoes
E_v:	Number of exposed mosquitoes
I_v:	Number of infectious mosquitoes
N_h:	Total human population size
N_v:	Total mosquito population size

Table S3: Parameters for the model (0.1) and their dimensions.

H_0:	Stable population size of humans. Humans.
Ψ_h:	Per capita birth rate of humans. We assume that $\Psi_h = \mu_h$ and the human population is at equilibrium. Time^{-1}.
Ψ_v:	Per capita recruitment rate of mosquitoes. Time^{-1}.
σ_v:	Number of times one mosquito would bite a human per unit time, if humans were freely available. This is a function of the mosquito's gonotrophic cycle (the amount of time a mosquito requires to produce eggs) and its preference for human blood. Time^{-1}.
σ_h:	The maximum number of mosquito bites a human can sustain per unit time. This is a function of the human's exposed surface area and any vector control interventions in place to reduce exposure to mosquitoes. Time^{-1}.
β_{hv}:	Probability of pathogen transmission from an infectious mosquito to a susceptible human given that a contact between the two occurs. Dimensionless.
β_{vh}:	Probability of pathogen transmission from an infectious human to a susceptible mosquito given that a contact between the two occurs. Dimensionless.
ν_h:	Per capita rate of progression of humans from the exposed state to the infectious state. $1/\nu_h$ is the average duration of the latent period. Time^{-1}.
ν_v:	Per capita rate of progression of mosquitoes from the exposed state to the infectious state. $1/\nu_v$ is the average duration of the extrinsic incubation period. Time^{-1}.
γ_h:	Per capita recovery rate for humans from the infectious state to the recovered state. $1/\gamma_h$ is the average duration of the infectious period. Time^{-1}.
μ_h:	Per capita death (and emigration) rate for humans. Time^{-1}.
μ_v:	Density-independent death rate for mosquitoes. Time^{-1}.
K_v:	Carrying capacity of mosquitoes. Mosquitoes.
r_v:	Natural growth rate of mosquitoes with no density dependence. Time^{-1}
P_h:	Fraction of bloodmeals that are human. Dimensionless.
Q_d:	Total number of bites available from dead-end hosts ($\sigma_d N_d$). Animal $\cdot$ Time^{-1}

Day post-exposure and percent infectious data for all mosquitoes sampled would be needed. Our estimate based on [15] was a mean of 10.2 with a range of 4.5-17. We used information from the World Health Organization and literature describing outbreaks, introductions of Zika by travelers, or sexual transmission of Zika with enough detail to inform human incubation and infectious period

Table S4: The parameters for **Zika virus** (left) and **chikungunya** (right) with baseline, range and references. Time is in days unless otherwise specified. All mosquito-related parameters are for *Ae. albopictus*. We varied the parameters as uniform distributions with given ranges. Parameters marked with a * were **not** varied, but set at the baseline value.

Par	Base	Range	Reference	Par	Base	Range	Reference
**Zika**				**Chikungunya**			
$1/\nu_h$	6d	$3-12$	[5, 6, 7, 8, 9, 10, 11]	$1/\nu_h$	3d	$2-4$	[1]
$1/\gamma_h$	$7d$	$3-14$	[5, 6, 7, 8, 9, 10, 11, 12]	$1/\gamma_h$	6d	$3-7$	[1]
$*1/\mu_h$	70 yrs	$68-76$	[1]	$*1/\mu_h$	70 yrs	$68-76$	[1]
β_{hv}	0.35	$0.1-0.75$	[1, 8]	β_{hv}	0.33	$0.001-.54$	[1]
β_{vh}	0.31	$0.1-0.75$	[1, 8]	β_{vh}	0.33	$0.3-0.75$	[1]
$*\Psi_v$	0.24	$0.22-0.26$	[1]	$*\Psi_v$	0.24	$0.22-0.26$	[1]
$1/\sigma_v$	3.8d	$2.0-5.26$	[13, 14]	$1/\sigma_v$	3.8d	$2.0-5.26$	[13, 14]
$1/\nu_v$	10.2d	$4.5-17$	[8, 15]	$1/\nu_v$	7.2d	$3.2-12.6$	[1, 16]
$1/\mu_v$	18d	$10-35$	[1, 17, 18, 19, 20, 21]	$1/\mu_v$	18d	$10-35$	[1, 17, 18, 19, 20, 21]
σ_h	19	$0.1-50$	[1, 22]	σ_h	19	$0.1-50$	[1, 22]
P_h	0.5	$0-1$	[23, 24, 25, 26, 27]	P_h	0.5	$0-1$	[23, 24, 25, 26, 27]
K_v/H_0	2	$0.5-10$	[1, 8]	K_v/H_0	2	$0.5-10$	[1, 8]
**NYC** (high human density)				**PA** (medium human density)			
H_0	25000/mi^2			H_0	11000/mi^2		
**DC** (medium human density)				**ATL** (low human density)			
H_0	8000/mi^2			H_0	3000/mi^2		

estimates.

Ae. albopictus have bimodal daily feeding activities which peak in the morning at twilight and 2 hours before sunset [18, 17]. The survival of mosquitoes are key factors in their effective control and disease prevention; the daily survival probability of male and female *Ae. albopictus* mosquitoes in La Reunion Island have been estimated to be approximately 0.95 [19] which is substantially higher than the value of 0.77 reported in for *Ae. albopictus* by [20] and in field studies for *Ae. aegypti* [31].

In Gabon, researchers found that the newly invaded *Ae. albopictus* were most likely the vector primarily responsible for outbreaks of chikungunya, dengue and Zika viruses. Of all sampled mosquito species in their study, only *Ae. albopictus* pools tested positive for all three pathogens [32, 33, 34]. [33] also used human landing studies to estimate the number of bites per person per hour during peak *Ae. albopictus* activity times (morning and early evening). Number of bites per hour ranged from 0.2 to 15.7 with a higher mean (4.58) in the suburbs than in downtown Libreville (0.65). Our model used number of bites per person per day ranging from 0 to 4, which is reasonable based on these studies and the presumed lower biting rates in cities with high screen and AC use. [35, 36] performed a risk assessment for Italy and *Ae. albopictus* and found minimal risk for transmission there. They did, however, use low *Ae. albopictus*-human biting rates corresponding to each mosquito biting a human once every 11 days (range from 6-20 days between human bites). With higher human usage, this number will rise significantly. [25] found that in Lebanon 47% of *Ae. albopictus* bloodmeals were on humans while other studies showed >50% or even 100% of blood meals on humans (e.g., [37]).

Researchers have recently computed R_0 for Zika using a range of methods and assumptions. It

is important to note that while some define R_0 for vector-borne disease as we have here, (method A $R_0 = \sqrt{R_{hv} R_{vh}}$) or the number of secondary infections in one generation (i.e. human to mosquito or mosquito to human), others define it as (method B $R_0 = R_{hv} R_{vh}$) or the number of secondary cases in two generations (i.e. human to human or mosquito to mosquito). Thus, R_0 for method B is the square of R_0 for method A. [12] estimated a mean basic reproduction number of 3.1 on Yap island with a 95% confidence interval of (0.7,8.7) (method B). [38] computed an R_0 mean value of 4.5-5.8 in Yap Island with ranges from 2.8-12.5 (method A). In French Polynesia, [8] predicted mean R_0 values ranging from 1.9-3.1 with confidence ranges from (1.4-7.9) (method A). [38] predicted an R_0 mean of 1.8-2.0 in French Polynesia with ranges from 1.5-3.1 (method A). [39] computed an R_0 of 4.4 with ranges from (3.0-6.2) in Colombia (method B), while [40] predicted an R_0 value of 1.6-2.2 in Antioquia, Colombia (method B). [41] predicted R_0 mean of 4.82 (2.34,8.32) with traditional data sources in Colombia and mean of 2.56 (1.42,3.83) for their nontraditional internet data sources (method B). [42] estimated R_0 values ranging from <1 to 11.62 for different regions of South America (method B). In summary, our mean R_0 value (method A) for Zika in the eastern United States of 1.1 is reasonable in the context of past and current outbreaks in other regions.

References

[1] Carrie A Manore, Kyle S Hickmann, Sen Xu, Helen J Wearing, and James M Hyman. Comparing dengue and chikungunya emergence and endemic transmission in a. aegypti and a. albopictus. *Journal of theoretical biology*, 356:174–191, 2014.

[2] Louis D Bergsman, James M Hyman, and Carrie A Manore. A mathematical model for the spread of west nile virus in migratory and resident birds. *Mathematical biosciences and engineering: MBE*, 13(2):401–424, 2016.

[3] N. Chitnis, JM Cushing, and JM Hyman. Bifurcation analysis of a mathematical model for malaria transmission. *SIAM Journal on Applied Mathematics*, 67(1):24–45, 2006.

[4] Marjorie J Wonham, Mark A Lewis, Joanna Rencławowicz, and P Van den Driessche. Transmission assumptions generate conflicting predictions in host–vector disease models: a case study in West Nile virus. *Ecology Letters*, 9(6):706–725, 2006.

[5] Gubio S Campos, Antonio C Bandeira, and Silvia I Sardi. Zika virus outbreak, bahia, brazil. *Emerging infectious diseases*, 21(10):1885, 2015.

[6] Brian D Foy, Kevin C Kobylinski, Joy L Chilson Foy, Bradley J Blitvich, Amelia Travassos da Rosa, Andrew D Haddow, Robert S Lanciotti, and Robert B Tesh. Probable non-vector-borne transmission of zika virus, colorado, usa. *Emerg Infect Dis*, 17(5):880–2, 2011.

[7] Kevin Fonseca, Bonnie Meatherall, Danielle Zarra, Michael Drebot, Judy MacDonald, Kanti Pabbaraju, Sallene Wong, Patricia Webster, Robbin Lindsay, and Raymond Tellier. First case of zika virus infection in a returning canadian traveler. *The American journal of tropical medicine and hygiene*, 91(5):1035–1038, 2014.

[8] Adam J Kucharski, Sebastian Funk, Rosalind M Eggo, Henri-Pierre Mallet, W John Edmunds, and Eric J Nilles. Transmission dynamics of zika virus in island populations: a modelling analysis of the 2013–14 french polynesia outbreak. *PLoS Negl Trop Dis*, 10(5):e0004726, 2016.

[9] Justin Lessler, Cassandra T Ott, Andrea C Carcelen, Jacob M Konikoff, Joe Williamson, Qifang Bi, Nicholas G Reich, Derek AT Cummings, Lauren M Kucirka, and Lelia H Chaisson. Times to key events in the course of zika infection and their implications for surveillance: A systematic review and pooled analysis. *bioRxiv*, page 041913, 2016.

[10] G Venturi, L Zammarchi, C Fortuna, ME Remoli, E Benedetti, C Fiorentini, M Trotta, C Rizzo, A Mantella, G Rezza, et al. An autochthonous case of zika due to possible sexual transmission, florence, italy, 2014. *Euro Surveill*, 21(8):30148, 2016.

[11] World Health Organization Western Pacific Region. *Zika virus fact sheet*, 2016 (accessed March 22, 2016).

[12] Sebastian Funk, Adam J. Kucharski, Anton Camacho, Rosalind M. Eggo, Laith Yakob, and W. John Edmunds. Comparative analysis of dengue and zika outbreaks reveals differences by setting and virus. *bioRxiv*, 2016.

[13] H. Delatte, G. Gimonneau, A. Triboire, and D. Fontenille. Influence of temperature on immature development, survival, longevity, fecundity, and gonotrophic cycles of Aedes albopictus, vector of chikungunya and dengue in the Indian Ocean. *Journal of Medical Entomology*, 46(1):33–41, 2009.

[14] M.M. Sivanathan. *The Ecology And Biology Of Aedes Aegypti (L.) And Aedes Albopictus (Skuse)(Diptera: Culicidae) And The Resistance Status Of Aedes Albopictus (Field Strain) Against Organophosphates In Penang, Malaysia [QL536. M266 2006 f rb].* PhD thesis, Universiti Sains Malaysia, 2006.

[15] Pei-Sze Jeslyn Wong, Mei-zhi Irene Li, Chee-Seng Chong, Lee-Ching Ng, and Cheong-Huat Tan. Aedes (stegomyia) albopictus (skuse): a potential vector of zika virus in singapore. *PLoS Negl Trop Dis*, 7(8):e2348, 2013.

[16] Rebecca C Christofferson, Daniel M Chisenhall, Helen J Wearing, and Christopher N Mores. Chikungunya viral fitness measures within the vector and subsequent transmission potential. *PloS one*, 9(10):e110538, 2014.

[17] A Paulo G Almeida, Susana SSG Baptista, Carla AGCC Sousa, M Teresa LM Novo, Helena C Ramos, Nicholas A Panella, Marvin Godsey, M João Simões, M Luisa Anselmo, Nicholas Komar, et al. Bioecology and vectorial capacity of aedes albopictus (diptera: Culicidae) in macao, china, in relation to dengue virus transmission. *Journal of medical entomology*, 42(3):419–428, 2005.

[18] William A Hawley. The biology of aedes albopictus. *Journal of the American Mosquito Control Association. Supplement*, 1:1–39, Dec. 1988.

[19] R Lacroix, Hélène Delatte, T Hue, and P Reiter. Dispersal and survival of male and female aedes albopictus (diptera: Culicidae) on reunion island. *Journal of medical entomology*, 46(5):1117–1124, 2009.

[20] ML Niebylski and GB Craig Jr. Dispersal and survival of aedes albopictus at a scrap tire yard in missouri. *Journal of the American Mosquito Control Association*, 10(3):339–343, 1994.

[21] Joanna Waldock, Nastassya L Chandra, Jos Lelieveld, Yiannis Proestos, Edwin Michael, George Christophides, and Paul E Parham. The role of environmental variables on aedes albopictus biology and chikungunya epidemiology. *Pathogens and global health*, 107(5):224–241, 2013.

[22] N. Chitnis, J.M. Hyman, and J.M. Cushing. Determining important parameters in the spread of malaria through the sensitivity analysis of a mathematical model. *Bulletin of Mathematical Biology*, 70(5):1272–1296, 2008.

[23] Helene Delatte, Amelie Desvars, Anthony Bouétard, Séverine Bord, Geoffrey Gimonneau, Gwenaël Vourc'h, and Didier Fontenille. Blood-feeding behavior of aedes albopictus, a vector of chikungunya on la réunion. *Vector-Borne and Zoonotic Diseases*, 10(3):249–258, 2010.

[24] Ary Faraji, Andrea Egizi, Dina M Fonseca, Isik Unlu, Taryn Crepeau, Sean P Healy, and Randy Gaugler. Comparative host feeding patterns of the asian tiger mosquito, aedes albopictus, in urban and suburban northeastern usa and implications for disease transmission. *PLoS Negl Trop Dis*, 8(8):e3037, 2014.

[25] Nabil Haddad, Laurence Mousson, Marie Vazeille, Soulaima Chamat, Joelle Tayeh, Mike A Osta, and Anna-Bella Failloux. Aedes albopictus in lebanon, a potential risk of arboviruses outbreak. *BMC infectious diseases*, 12(1):300, 2012.

[26] Joaquín Muñoz, Roger Eritja, Miguel Alcaide, Tomás Montalvo, Ramón C Soriguer, and Jordi Figuerola. Host-feeding patterns of native culex pipiens and invasive aedes albopictus mosquitoes (diptera: Culicidae) in urban zones from barcelona, spain. *Journal of medical entomology*, 48(4):956–960, 2011.

[27] Laura Valerio, Francesca Marini, Gioia Bongiorno, Luca Facchinelli, Marco Pombi, Beniamino Caputo, Michele Maroli, and Alessandra della Torre. Host-feeding patterns of aedes albopictus (diptera: Culicidae) in urban and rural contexts within rome province, italy. *Vector-Borne and Zoonotic Diseases*, 10(3):291–294, 2010.

[28] Rebecca C. Christofferson, Daniel M. Chisenhall, Helen J. Wearing, and Christopher N. Mores. Chikungunya viral fitness measures within the vector and subsequent transmission potential. *PLoS ONE*, 9(10):1–8, 10 2014.

[29] Thais Chouin-Carneiro, Anubis Vega-Rua, Marie Vazeille, André Yebakima, Romain Girod, Daniella Goindin, Myrielle Dupont-Rouzeyrol, Ricardo Lourenço-de Oliveira, and Anna-Bella Failloux. Differential susceptibilities of aedes aegypti and aedes albopictus from the americas to zika virus. *PLoS Negl Trop Dis*, 10(3):e0004543, 2016.

[30] M Di Luca, F Severini, L Toma, D Boccolini, R Romi, ME Remoli, M Sabbatucci, C Rizzo, G Venturi, G Rezza, et al. Experimental studies of susceptibility of italian aedes albopictus to zika virus. *Euro Surveill.*, 21, 2016.

[31] Paul Reiter. Oviposition, dispersal, and survival in aedes aegypti: implications for the efficacy of control strategies. *Vector-Borne and Zoonotic Diseases*, 7(2):261–273, 2007.

[32] Gilda Grard, Mélanie Caron, Illich Manfred Mombo, Dieudonné Nkoghe, Statiana Mboui Ondo, Davy Jiolle, Didier Fontenille, Christophe Paupy, and Eric Maurice Leroy. Zika virus in gabon (central africa)–2007: a new threat from aedes albopictus? *PLoS Negl Trop Dis*, 8(2):e2681, 2014.

[33] C. Paupy, B. Ollomo, B. Kamgang, S. Moutailler, D. Rousset, M. Demanou, J.P. Hervé, E. Leroy, and F. Simard. Comparative role of Aedes albopictus and Aedes aegypti in the emergence of dengue and chikungunya in Central Africa. *Vector-Borne and Zoonotic Diseases*, 10(3):259–266, 2010.

[34] Christophe Paupy, Fabrice Kassa Kassa, Mélanie Caron, Dieudonné Nkoghé, and Eric M Leroy. A chikungunya outbreak associated with the vector Aedes albopictus in remote villages of Gabon. *Vector-Borne and Zoonotic Diseases*, 12(2):167–169, 2012.

[35] Giorgio Guzzetta, Fabrizio Montarsi, Frédéric Alexandre Baldacchino, Markus Metz, Gioia Capelli, Annapaola Rizzoli, Andrea Pugliese, Roberto Rosà, Piero Poletti, and Stefano Merler. Potential risk of dengue and chikungunya outbreaks in northern italy based on a population model of aedes albopictus (diptera: Culicidae). *PLOS Negl Trop Dis*, 10(6):e0004762, 2016.

[36] G Guzzetta, P Poletti, F Montarsi, F Baldacchino, G Capelli, A Rizzoli, R Rosà, and S Merler. Assessing the potential risk of zika virus epidemics in temperate areas with established aedes albopictus populations. *Euro surveillance: bulletin Europeen sur les maladies transmissibles= European communicable disease bulletin*, 21(15), 2016.

[37] Basile Kamgang, Elysée Nchoutpouen, Frédéric Simard, and Christophe Paupy. Notes on the blood-feeding behavior of aedes albopictus (diptera: Culicidae) in cameroon. *Parasites & vectors*, 5(1):1, 2012.

[38] Hiroshi Nishiura, Ryo Kinoshita, Kenji Mizumoto, Yohei Yasuda, and Kyeongah Nah. Transmission potential of zika virus infection in the south pacific. *International Journal of Infectious Diseases*, 45:95–97, 2016.

[39] Sherry Towers, Fred Brauer, Carlos Castillo-Chavez, Andrew KI Falconar, Anuj Mubayi, and Claudia ME Romero-Vivas. Estimation of the reproduction number of the 2015 zika virus outbreak in barranquilla, colombia, and a first estimate of the relative role of sexual transmission. *arXiv preprint arXiv:1606.01422*, 2016.

[40] Gerardo Chowell, Doracelly Hincapie-Palacio, Juan Ospina, Bruce Pell, Amna Tariq, Sushma Dahal, Seyed Moghadas, Alexandra Smirnova, Lone Simonsen, and Cécile Viboud. Using phenomenological models to characterize transmissibility and forecast patterns and final burden of zika epidemics. *PLOS Currents Outbreaks*, 2016.

[41] Maimuna S Majumder, Mauricio Santillana, Sumiko R Mekaru, Denise P McGinnis, Kamran Khan, and John S Brownstein. Utilizing nontraditional data sources for near real-time estimation of transmission dynamics during the 2015-2016 colombian zika virus disease outbreak. *JMIR Public Health and Surveillance*, 2(1):e30, 2016.

[42] Alex Perkins, Amir Siraj, Corrine Warren Ruktanonchai, Moritz Kraemer, and Andrew Tatem. Model-based projections of zika virus infections in childbearing women in the americas. *bioRxiv*, page 039610, 2016.
